# Evaluation of the Sensitivity and Specificity of the Modified Moenchengladbach Resuscitation Team Activation (M^2^-GRETA) Criteria for Streamlined Resuscitation Room Activation at the Time of Initial Emergency Department Assessment

**DOI:** 10.3390/jcm15135221

**Published:** 2026-07-03

**Authors:** Ruth-Nardin Dorsten, Sebastian Bergrath, Jessika Stefanie Kreß, Marc Deussen, Jana Vienna Rödler

**Affiliations:** 1 Center of Clinical Acute and Emergency Medicine, Kliniken Maria Hilf Moenchengladbach, Academic Teaching Hospital of RWTH Aachen University, 41063 Moenchengladbach, Germany; ruth.brunecker@web.de (R.-N.D.); j.kress@asklepios.com (J.S.K.); janavienna.roedler@mariahilf.de (J.V.R.); 2 Medical Faculty of RWTH, Aachen University, 52074 Aachen, Germany; 3 Emergency Department, Asklepios Klinik St. Georg, 20099 Hamburg, Germany; 4 City of Moenchengladbach, Emergency Medical Service, Medical Direction, 41061 Moenchengladbach, Germany; marc.deussen@moenchengladbach.de

**Keywords:** M^2^-GRETA criteria, resuscitation room activation, non-trauma patients, trauma patients, emergency department triage

## Abstract

**Background**: Detailed criteria for resuscitation room activation exist for trauma and non-trauma patients in national recommendations and guidelines. By consolidation and grouping these parameters, we developed the modified Moenchengladbach Resuscitation Team Activation (M^2^-GRETA) criteria to create a unified and simplified activation system for both patient groups. This study evaluated the sensitivity and specificity of M^2^-GRETA during initial assessment in the emergency department (ED). **Methods**: This retrospective study (EK 24-135) included 1591 patients treated in the ED of a 754-bed teaching hospital. Patients were assigned either to the resuscitation room group (14 December 2022–28 August 2023) or the observation unit control group (1 January–4 February 2024). Exclusion criteria were age <18 yrs, inter-hospital transfers, or non-resuscitation patients requiring intensive care unit (ICU)/stroke unit admission within 24 h. Both groups were assessed for the presence of M^2^-GRETA criteria. **Results**: Mortality was higher in resuscitation patients than controls (22.5% vs. 4.8%, *p* < 0.001), and 62.3% required ICU transfer. In non-trauma patients, M^2^-GRETA showed a sensitivity of 96.3% and a specificity of 89.3%. In trauma patients, it showed a sensitivity of 87.5% and a specificity of 75.0%. Positive M^2^-GRETA criteria were more common in resuscitation room patients than among controls (non-trauma: 96.3% vs. 10.7%; trauma: 87.5% vs. 25.0%). The mean numbers of positive criteria in the resuscitation room groups were as follows: non-trauma 2.9 vs. 0.1; trauma 1.4 vs. 0.3; *p* < 0.001. Likelihood ratios (LR) indicated higher diagnostic utility in non-trauma patients (LR + 8.92; LR − 0.04) than in trauma patients (LR + 3.50; LR − 0.17). **Conclusions**: The M^2^-GRETA criteria display a promising diagnostic utility in identifying patients requiring resuscitation room activation, particularly among non-trauma cases, supporting the early recognition of critically ill patients and optimized triage. Yet these preliminary results should be subject to future validation via prospective multicenter studies.

## 1. Introduction

National recommendations and clinical guidelines provide detailed criteria for the activation of resuscitation room activation in both trauma and non-trauma patients [1,2]. The structured management of critically ill patients remains a key challenge in contemporary emergency medicine. The emergency department (ED) represents the pivotal interface between prehospital emergency care and in-hospital acute management, where rapid decision-making and coordinated interdisciplinary processes are essential. Timely and appropriate activation of the resuscitation room team is essential to ensure that specialized personnel, diagnostic resources, and technical infrastructure are available and has been shown to be closely associated with patient positive outcomes [3,4,5,6].

Delayed or insufficient activation may result in the postponement of diagnostic and therapeutic interventions, potentially negatively affecting patient outcomes. However, both undertriage and overtriage in resuscitation room activation are associated with relevant disadvantages [7,8]. For example, excessive activation may result in substantial personnel workload, alarm fatigue, and increased consumption of limited resources. Consequently, the development of activation criteria must achieve an appropriate balance between sensitivity and specificity, which is essential to ensure high-quality emergency care.

In trauma care, resuscitation room activation has become increasingly standardized over recent decades [2,3]. National and international trauma registries, along with evidence-based guidelines such as the German S3 guideline for the management of severely injured patients, provide well-defined activation criteria based on injury mechanisms, injury patterns, and physiological parameters [2,3]. These structured approaches have contributed to improved process quality and outcomes in trauma patients. In contrast, comparable standardized and validated activation criteria for critically ill non-trauma patients are less well established [9,10,11]. The German White Paper on the care of critically ill patients in EDs provides recommendations on structural and organizational requirements, equipment, and documentation; however, it does not provide specific, validated criteria for resuscitation room activation. In contrast, the “V_2_iSiOn” alert criteria are based on relevant vital signs and were developed to identify non-trauma patients who may require resuscitation room care [12].

The Moenchengladbach Resuscitation Team Activation (M^2^-GRETA) criteria were designed to provide a unified framework for resuscitation room activation or capacity reallocation for both non-trauma and trauma patients. The objective of this study was to evaluate the diagnostic performance of the M^2^-GRETA criteria and to assess their predictive validity for clinically relevant outcomes in critically ill non-trauma and trauma patients.

## 2. Materials and Methods

### 2.1. Study Design

This retrospective, exploratory cohort study was conducted between 14 December 2022 and 4 February 2024 and evaluated the presence of M^2^-GRETA in non-trauma and trauma patients at the time they arrived in the ED.

The aim of the study was to assess the ability of these newly developed criteria to predict if a resuscitation room activation was needed. The sensitivity and specificity of individual criteria as well as their combined performance were analyzed to determine how effective they were in identifying patients requiring immediate care while minimizing overtriage.

### 2.2. Study Population

Patients were eligible for inclusion if they presented to the ED and were subsequently later admitted to a regular ward, intensive care unit (ICU), stroke unit, or chest pain unit (CPU) or died in the resuscitation area during the study period.

Importantly, the M^2^-GRETA criteria were not available to treating clinicians and were not used to inform resuscitation room allocation or any other clinical decision-making process. Allocation to the resuscitation room and the performance of resuscitation room interventions were determined prospectively in real time according to institutional trauma team activation protocols and the clinical judgement of the attending emergency physicians. Thus, the reference process reflects standard clinical practice independent of the M^2^-GRETA score. For the purpose of this study, we subsequently assessed whether patients who received resuscitation room care and interventions would have been identified as M^2^-GRETA positive based on retrospective calculation.

### 2.3. M^2^-GRETA Criteria

The M^2^-GRETA criteria were systematically derived by condensing criteria from the German White Paper and the German S3 guideline for the management of severely injured patients. A local expert committee conducted three structured rounds of discussion and decision-making to reach consensus and ensure that the final criteria reflected both guideline recommendations and local clinical expertise.

### 2.4. Group Classification

This study included two patient cohorts: a control group and a resuscitation room group.

The control group comprised all adult patients presenting to the ED who were considered clinically stable and eligible for admission to a regular ward. Patients were excluded from the control group if they were minors (<18 years), were transferred from another hospital, required transfer to the ICU, or were admitted to a stroke unit or CPU.

The resuscitation room group consisted of all patients who underwent at least one invasive intervention during their initial management in the resuscitation room. Patients were excluded from this group if no invasive procedure was performed.

Invasive and other resuscitation room criteria were defined as follows:

- Arterial line placement
- Central venous catheter (CVC) insertion/central venous access
- Chest tube insertion
- Endotracheal intubation (ETI)
- Non-invasive ventilation (NIV)
- Emergency electrical cardioversion (eCV)
- Activation of massive transfusion protocol (“hotline”)
- Emergency endoscopy in the resuscitation room (esophagogastroduodenoscopy/colonoscopy)
- Emergency imaging (computed tomography (CT)/magnetic resonance imaging) performed directly from the resuscitation room
- Bronchoscopy
- Coagulation management (targeted hemostatic therapy)
- Mechanical thrombectomy
- Direct transfer to the operating room
- Direct transfer to an external hospital with specialized care (neurosurgery, cardiothoracic surgery, obstetrics)
- Administration of catecholamines
- Cardiopulmonary resuscitation
- Application of tourniquets
- Sedation

### 2.5. Assessment of M^2^-GRETA Criteria

The M^2^-GRETA criteria were assessed retrospectively based on structured review of electronic medical records.

For each patient, all relevant clinical documentation, including triage notes, vital parameters, laboratory findings, imaging reports, procedural documentation, and discharge summaries, were reviewed. The presence of predefined M^2^-GRETA criteria was assessed at the time of initial presentation in the ED.

Patients were retrospectively classified according to whether they fulfilled M^2^-GRETA criteria, thus warranting resuscitation room activation. Criteria were considered met if at least one documented parameter or intervention corresponded to the predefined M^2^-GRETA indicators. In cases of ambiguity, classification was based on consensus review to ensure consistent application of the criteria.

All data were extracted from routinely collected medical records. An electronic health record system (iMedOne, Deutsche Telekom Healthcare and Security Solutions GmbH, Bonn, Germany) served as the primary data source. In addition, all paper-based medical records were systematically reviewed. Study data were transferred into a secured, access-restricted Excel database (Microsoft Corp., Redmond, WA, USA) and were secondarily pseudonymized.

### 2.6. Local Setting

Our academic teaching hospital (Kliniken Maria Hilf, Moenchengladbach, Germany) comprises 754 beds, including 45 ICU beds, an 18-bed supraregional stroke unit, and a level II ED. The hospital is certified as an oncologic center, a cardiac arrest center, and a regional trauma center (level II). The core ED team consists of consultant physicians, specialists, and resident physicians in training.

### 2.7. Outcomes

Patients in the control group were enrolled between 1 January 2024, and 4 February 2024, while patients in the resuscitation room group were included between 14 December 2022 and 28 August 2023. Both groups were systematically evaluated according to the M^2^-GRETA criteria.

Outcome measures included length of stay in the ED, mode of arrival (referral by general practitioner, emergency medical service (EMS), prehospital EMS-physician, or walk-in).

**Figure 1:**
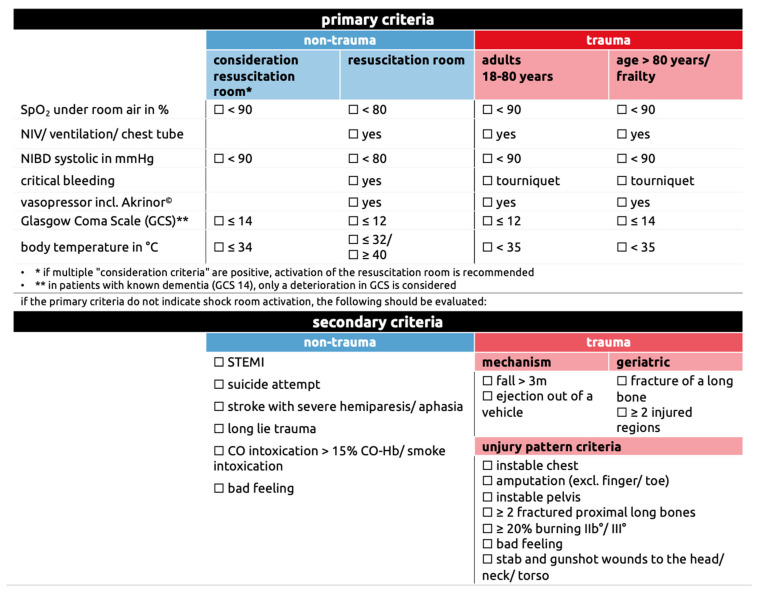
Template for daily use of the M^2^-GRETA criteria.

As shown in Figure 1 the primary criteria were categorized according to the ABCDE approach [13,14]. Vital signs measured at presentation in the ED included peripheral oxygen saturation on room air, blood pressure, given catecholamines (including cafedrine/theodrenaline), Glasgow Coma Scale (GCS) score, and body temperature. The respiratory rate was deliberately excluded, as it is frequently estimated rather than objectively measured in routine clinical practice, which may limit its reliability and reproducibility.

If the primary criteria were not fulfilled, additional secondary criteria, such as specific clinic conditions, were considered to support triage decisions and ensure appropriate allocation of emergency resources. One such condition is long lie, in which a patient remains immobilized for a prolonged period after a fall or acute event. Long-lie trauma was defined as a condition resulting from prolonged, involuntary immobilization lasting several hours to days. It is typically associated with complications such as pressure ulcers, rhabdomyolysis, acute kidney injury, hypothermia, electrolyte disorders, and dehydration [15].

The M^2^-GRETA criteria differ between adult and geriatric populations regarding trauma patients. In the geriatric cohort, patients were classified according to the M^2^-GRETA criteria if they were older than 80 years or exhibited a frailty level of ≥5 on the Clinical Frailty Scale [16]. The presence of critical bleeding was recorded in non-trauma and trauma patients. Mortality was recorded both in the ED and during the subsequent hospital stay.

### 2.8. Ethics Approval and Consent to Participate

This retrospective study was conducted in accordance with the principles of the Declaration of Helsinki. An ethics application was submitted to the responsible ethics committee (Medical Faculty of RWTH Aachen University, Germany) and approved on 15 April 2024 (registration number EK 24-135). Additionally, the study was registered at the Clinical Trial Center of RWTH Aachen University, Germany (CTC-A Number 24-181), before it began. In accordance with national regulations, informed consent was not required for this type of study. The requirement for informed consent was waived by the responsible ethics committee as part of the approval process.

### 2.9. Statistics

Descriptive data were presented as mean with standard deviation (SD) and median with interquartile ranges (IQR). We compared admission characteristics between both groups using the Chi-squared test were applicable. Given the exploratory character of the study, *p*-values < 0.05 were considered to be statistically significant. Group comparisons for continuous variables were performed using the Welch’s *t*-test for normally distributed data. Categorical variables were compared using Fisher’s exact test. For the estimation of proportions, 95% confidence intervals (CI) were calculated using the Wilson score method. This approach was chosen due to its improved performance over the standard normal approximation, particularly for proportions close to 0 or 1 and in samples with varying group sizes. All statistical analyzes were carried out with Prism 8.4.2 GraphPad Software, San Diego, CA, USA.

## 3. Results

### 3.1. Demographic Baseline Data of the Study

**Figure 2:**
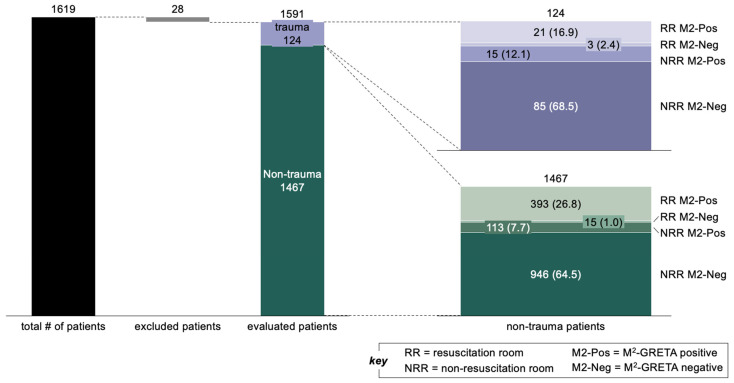
Split of total to evaluated patients as well as (non-)trauma patients.

During the study period, 1591 patients were included. A total of 28 patients were excluded from further analysis because data were either incomplete or missing. In the resuscitation room, 432 patients were treated and assigned to the resuscitation room group, whereas 1159 patients were not treated in the resuscitation room and were consequently assigned to the control group (Figure 2). In the resuscitation room cohort, 97 of 432 patients (22.5%) died, whereas 57 of 1159 patients (4.8%) in the control group died during the hospital stay (*p* < 0.001).

Overall, 269 of 364 patients (62,3%) were directly transferred from the ED to the ICU (resuscitation room group). Trauma patients treated in the resuscitation room had a mean frailty score of 3.6 (SD ± 2), whereas trauma patients not treated in the resuscitation room had a mean frailty score of 4.1 (SD ± 2) (*p* = 0.28).

The demographic baseline data for the non-trauma and trauma patient groups are presented in Table 1 and Table 2. Among non-trauma patients, no significant differences were observed between the resuscitation room and control groups with respect to the average age in years or average patient height (Table 1). Similarly, no significant difference in age was observed between patients treated in the resuscitation room and control groups (Table 2).

The proportion of M^2^-GRETA-positive cases among non-trauma patients in the resuscitation room group was 96.3% (95% CI 94.0–97.8), based on Wilson score estimation. In the non-trauma control group, 10.7% of patients were M^2^-GRETA positive (95% CI 8.9–12.7), also estimated using the Wilson score method.

Among trauma patients in the resuscitation room group, 21 of 24 patients (87.5%) fulfilled the M^2^-GRETA criteria. The estimated proportion of M^2^-GRETA-positive cases was 87.5% (95% CI 69.0–95.7%), based on Wilson score estimation. In the trauma control group, 25 of 100 patients (25.0%) were M^2^-GRETA positive, corresponding to an estimated proportion of 25.0% (95% CI 17.6–34.3%).

### 3.2. Sensitivity and Specificity

The M^2^-GRETA criteria demonstrated a sensitivity of 96.3% and a specificity of 89.3% in non-trauma patients. Among trauma patients, sensitivity and specificity were 87.5% and 75.0%, respectively.

At least one positive M^2^-GRETA criterion was identified in 393 of 408 patients in the non-trauma resuscitation group, which corresponds to a prevalence of 96.3%. In contrast, 113 of 1059 patients in the non-trauma control group presented with at least one positive M^2^-GRETA criterion, yielding a prevalence of 10.7%. Among trauma patients, 21 of 24 individuals (87.5%) in the resuscitation group exhibited at least one positive M^2^-GRETA criterion, whereas 25 of 100 (25.0%) patients were positive in the trauma control group.

Regarding the prevalence of individual criteria, a Glasgow Coma Scale (GCS) score <15 was the most common finding among non-trauma patients treated in the resuscitation room (*n* = 233; 57.1%). In contrast, a GCS < 15 was observed in 55 non-trauma patients (5.2%) not treated in the resuscitation room.

Among trauma patients treated in the resuscitation room, geriatric patients with a GCS < 14 accounted for 5 out of 24 cases (20.8%). Among trauma patients not treated in the resuscitation room, the most frequent criterion was a fracture of a long tubular bone (*n* = 13; 13.0%) in a geriatric patient.

The mean number of positive M^2^-GRETA criteria differed markedly between the groups. Among non-trauma patients treated in the resuscitation room, the mean number of positive criteria was 2.9 (standard deviation (SD) 1.7) compared with a mean of 0.1 (SD 0.4) (*p* < 0.001) in non-trauma patients not treated in the resuscitation room. Among trauma patients, the mean number of positive criteria was 1.4 (SD 1.4) in the resuscitation room group, compared with 0.3 (SD 0.6) in trauma patients not treated in the resuscitation room (*p* < 0.001).

As shown in Table 3, all criteria except CO intoxication/smoke intoxication differed significantly between the non-trauma patients treated in and outside the resuscitation room.

In contrast, significant differences were observed in 9 out of 20 criteria in the trauma patient group (see Table 4).

Among non-trauma patients, the positive likelihood ratio was 8.92, and the negative likelihood ratio was 0.04. In trauma patients, these values were 3.50 and 0.17, respectively. For a GCS ≤12, the positive likelihood ratio was 30.28 in non-trauma patients and 12.50 in trauma patients.

## 4. Discussion

In this study, we evaluated the diagnostic performance of the M^2^-GRETA criteria in both trauma and non-trauma patients presenting to the ED, while focusing on the ability to identify patients requiring resuscitation room activation and ICU admission. To the best of our knowledge, this is the first study to report these findings in a patient cohort including both trauma and non-trauma patients.

To date, no internationally established criteria provide a unified approach to resuscitation room activation for both non-trauma and trauma patients [3,17,18]. Although numerous countries, including the United States and the United Kingdom, have well-defined trauma team activation protocols based on physiological, anatomical, and mechanism-of-injury criteria, these systems are generally limited to trauma patients. Critically ill non-trauma patients—such as those with out-of-hospital cardiac arrest, hemorrhagic shock, or acute cardiac decompensation—are usually managed through separate resuscitation or rapid response team protocols. This separation reflects both historical development and differences in clinical workflow, but it highlights a gap in standardized, integrated activation criteria for critically ill patients entering the resuscitation room. Closing this gap could streamline the identification and management for all such patients. The M^2^-GRETA criteria were developed to create a single unified framework that reflects non-trauma as well as trauma triggers. While the selected set of criteria needs to be subject to future validation, it represents the first take to address the current lack of such unified methodology. Our findings revealed a higher prevalence of positive M^2^-GRETA criteria among patients admitted to the resuscitation room compared with controls, supporting the potential utility of the criteria in identifying patients at risk of critical illness. Specifically, 96.3% of non-trauma and 87.5% of trauma patients in the resuscitation room cohort fulfilled at least one M^2^-GRETA criterion, compared with 10.7% and 25.0% in the respective control groups. These findings are consistent with previous observations in German non-trauma cohorts, such as the OBSERvE and OBSERvE-DUS studies by Bernhard et al., which highlighted the complexity and high severity of illness among non-trauma resuscitation room patients and the need for structured triage protocols. Compared with other alert systems, such as the “V_2_iSiOn” alert criteria, which primarily rely on vital sign abnormalities in non-trauma patients, M^2^-GRETA extends this approach by incorporating additional clinical indicators and is applicable to both trauma and non-trauma populations [12]. This broader scope may contribute to its high sensitivity, particularly among non-trauma patients who often present with subtle but clinically significant abnormalities. Critically ill non-trauma patients represent a relevant subgroup of ED admissions requiring structured resuscitation room activation and interdisciplinary care. This patient population has been increasingly characterized in recent years, particularly through observational studies from German EDs [10,11].

Whereas structured algorithms for trauma resuscitation have been well established for decades, comparably structured approaches for critically ill non-trauma patients have only recently emerged [19].

The M^2^-GRETA test exhibited high sensitivity and specificity, particularly in non-trauma patients (sensitivity 96.3%, specificity 89.3%). The positive and negative likelihood ratios further underscore its diagnostic utility, with LR+ of 8.92 and LR– of 0.04, respectively, in non-trauma patients. These findings indicate that a positive test result substantially increases the probability that resuscitation room care is required, while a negative result effectively rules it out. Although the LR+ was lower (3.50) and LR– higher (0.17) in trauma patients, the test still provides valuable guidance for triage decisions. The markedly higher positive likelihood ratio associated with a GCS ≤ 12, particularly in non-trauma patients, highlights the strong predictive value of relevant neurological impairment for the need of resuscitation room activation and underlines the importance of early neurological assessment during initial triage in the ED. No significant difference in frailty scores was observed between trauma patients treated in and outside the resuscitation room and those managed outside the resuscitation room, suggesting that frailty alone may not be a decisive factor in determining the need for resuscitation room activation in this patient population. However, we cannot draw any definitive conclusion for trauma patients, as the trauma patient cohort was very small.

Furthermore, the width of the CI differed considerably between trauma and non-trauma patients, primarily reflecting differences in sample size. Whereas the wider interval in the trauma cohort indicates greater statistical uncertainty, the overall pattern remained consistent across both patient populations. Importantly, the CIs of the resuscitation room groups were clearly separated from those of the respective control groups, supporting the stability and clinical relevance of the observed findings.

Our analysis of individual M^2^-GRETA criteria revealed that altered mental status, reflected in a GCS < 15, was the most frequent finding among non-trauma patients in the resuscitation room, whereas among trauma patients, geriatric status with specific injuries predominated. These findings align with prior studies emphasizing the importance of neurological compromise and frailty in predicting critical illness as well as the need for intensive monitoring in the ED. Furthermore, the mean number of positive criteria was significantly higher in resuscitation room patients compared with controls in both trauma and non-trauma groups, thus supporting the cumulative value of multiple criteria in clinical triage.

The high mortality rate in the resuscitation room cohort (22.5% vs. 4.8% in controls) is consistent with the greater acuity of illness in these patients and supports the need for timely identification of patients at increased risk of adverse outcomes. These findings support the hypothesis that the M^2^-GRETA criteria may be a promising framework to support decision-making regarding initial patient management and resource allocation in the ED.

Importantly, structured triage and activation algorithms have improved early identification and allocation of critical resources when they are implemented in trauma care through Advanced Trauma Life Support-based primary surveys and the German S3 guideline for the management of severely injured patients. Our findings suggest that a similarly structured approach to non-trauma patients may aid in the recognition of critically ill individuals; however, its potential impact on undertriage and resuscitation room activation should be investigated by future prospective studies.

In conclusion, within the limitations of this study, the M^2^-GRETA criteria displayed promising diagnostic utility for identifying patients who require resuscitation room activation, particularly among non-trauma patients. Incorporating these criteria into ED triage may improve early recognition of critically ill patients, optimize resource utilization, and potentially improve patient outcomes. Future prospective multicenter studies are needed to validate these findings and further refine the criteria for trauma populations.

### Limitations

Several limitations of this study should be acknowledged.

First, this was a single center study conducted in a German emergency department, which may limit the generalizability of the findings to other healthcare systems with different ED structures, triage processes, or resuscitation room organization.

Second, the retrospective design is subject to inherent limitations, including potential issues related to data completeness and accuracy. Although the M^2^-GRETA score was calculated independently and retrospectively, some overlap exists between clinical variables used in routine resuscitation room activation and those included in the M^2^-GRETA criteria. This may introduce a degree of spectrum or incorporation-related bias when interpreting the agreement between both approaches. Nevertheless, resuscitation room allocation was based on established institutional trauma team activation protocols and real-time clinical judgement and was not influenced by the M^2^-GRETA score.

Third, differences in sampling periods between study groups represent a potential limitation. Because resuscitation room admissions occur less frequently, a longer inclusion period was required to achieve an adequate sample size, whereas the control cohort was assembled from a shorter time window. As a result, the groups were not strictly contemporaneous, which may affect comparability.

Fourth, the composition of the control group should be considered when interpreting the results. Patients admitted to the ICU, stroke unit, or CPU were excluded, resulting in a control group primarily consisting of patients treated on regular wards. This may limit the representativeness of higher-acuity ED patients who did not require resuscitation room activation and could influence estimates of diagnostic performance.

Fifth, the relatively small number of trauma patients in the resuscitation cohort may have reduced the precision of subgroup estimates, including sensitivity, specificity, and likelihood ratios. Therefore, trauma-specific results should be interpreted as exploratory and require validation in larger prospective cohorts.

Finally, while M^2^-GRETA displayed promising diagnostic utility, its potential impact on clinical workflow parameters such as time to intervention, undertriage rates, overtriage rates, and resource utilization remains speculative and should be evaluated in future prospective implementation studies. In addition, successful translation into clinical practice will require integration with clinical judgement, existing triage algorithms, and structured implementation within EMS workflows.

## 5. Conclusions

Within the limitations of this study, the M^2^-GRETA criteria provide a promising tool for identifying patients who require resuscitation room activation, with particularly good performance in non-trauma cases. Implementing them in the ED may improve a physician’s ability to recognize critically ill patients earlier, while also supporting more effective triage and resource allocation. Further multicenter studies, especially for trauma populations, are warranted to confirm, validate, and refine their clinical utility.

## Figures and Tables

**Table 1:** Demographic baseline data from non-trauma patients.

Demographics				
Parameter	∑	Resuscitation Room Group	Control Group	*p*-Value
*n*	1467	408	1059	
gender distribution m/f/d (%)	791/676/0 (53.9/46.1/0)	235/173/0 (57.6/42.4/0)	556/503/0 (52.5/47.5/0)	0.08
average age in years ± SD	66 ± 18	67 ± 16	65 ± 19	0.04
average weight in kg ± SD (min/max)	80 ± 22 (35/270)	83 ± 28 (37/270)	79 ± 20 (35/190)	<0.01
average height in cm ± SD (min/max)	171 ± 10 (142/204)	171 ± 10 (142/204)	171 ± 10 (145/200)	1.00
mean BMI ± SD (min/max)	27 ± 7 (13/83)	28 ± 9 (14/83)	27 ± 6 (13/61)	0.03
M^2^-GRETA positive (%)	506 (34.3)	393 (96.3)	113 (10.7)	<0.01

Key: m, male; f, female; d, diverse; M, mean; SD, standard deviation; BMI, Body Mass Index. Categorical variables were analyzed using Welch’s test and Chi-squared test.

**Table 2:** Demographic baseline data from trauma patients.

Demographics				
Parameter	∑	Resuscitation Room Group	Control Group	*p*-Value
*n*	124	24	100	
gender distribution m/f/d (%)	56/68/0 (45.2/54.8/0)	18/6/0 (75/25/0)	38/62/0 (38/62/0)	0.001
average age in years ± SD	69 ± 20	65 ± 17	70 ± 20	0.22
average weight in kg ± SD (min/max)	80 ± 21 (45/145)	94 ± 21 (64/145)	77 ± 19 (45/140)	0.001
average height in cm ± SD (min/max)	171 ± 10 (150/199)	179 ± 8 (168/192)	169 ± 10 (150/199)	<0.0001
mean BMI ± SD (min/max)	27 ± 5 (17/41)	28 ± 3 (22/34)	26 ± 5 (17/41)	0.01
M^2^-GRETA positive (%)	46 (37.1)	21 (87.5)	25 (25)	<0.001

Key: m, male; f, female; d, diverse; M, mean; SD, standard deviation; BMI, Body Mass Index. Categorical variables were analyzed using Fisher exact test and Welch’s test.

**Table 3:** Number and percentage of non-trauma patients meeting each M^2^-GRETA criterion:

Criteria Non-Trauma Patients	Resuscitation Room Group (*n*)	%	Control Group (*n*)	%	*p*-Value
primary criteria					
SpO_2_ under room air					
<90%	148	36.3	50	4.7	<0.001
<80%	66	6.2	7	0.7	<0.001
NIV	110	27.0	0	0.0	<0.001
ventilation	160	15.1	0	0.0	<0.001
chest tube	5	1.2	0	0.0	0.002
NIBD systolic					
<90 mmHg	52	12.7	8	0.8	<0.001
<80 mmHg	33	3.1	3	0.3	<0.001
vasopressor use incl. cafedrine/theodrenaline	149	36.5	0	0.0	<0.001
GCS					
≤14	233	57.1	55	5.2	<0.001
≤12	175	42.9	15	1.4	<0.001
body temperature					
≤34 °C	8	2.0	1	0.1	<0.001
≤32 °C	5	0.5	1	0.1	0.008
≥40 °C	4	1.0	1	0.1	0.022
secondary criteria					
STEMI	17	4.2	0	0.0	<0.001
suicide attempt	7	0.7	0	0.0	<0.001
stroke with severe hemiparesis/aphasia	32	7.8	6	0.6	<0.001
long lie trauma	5	1.2	3	0.3	0.041
CO intoxication > 15%/smoke intoxication	0	0.0	0	0.0	1.0
bad feeling	10	2.5	0	0.0	<0.001

Key: NIV = non-invasive ventilation, GCS = Glasgow Coma Scale, NIBD = non-invasive blood pressure, STEMI = ST segment elevation myocardial infarction; categorical variables were analyzed using Fisher’s test.

**Table 4:** Number and percentage of trauma patients meeting each M^2^-GRETA criterion:

Criteria Trauma Patients	Resuscitation Room Group (*n*)	%	Control Group (*n*)	%	*p*-Value
primary criteria					
SpO_2_ under room air					
<90%	0	0.0	0	0.0	1.0
NIV	0	0.0	0	0.0	1.0
ventilation	4	16.7	0	0.0	0.001
chest tube	1	4.2	0	0.0	0.18
NIBD systolic					
<90 mmHg	0	0.0	0	0.0	1.0
vasopressor use incl. cafedrine/theodrenaline	2	8.3	0	0.0	0.04
GCS					
geriatrics <14	5	20.8	3	3.0	0.01
<12	3	12.5	1	1.0	0.01
body temperature					
<35 °C	0	0.0	1	1.0	1.0
secondary criteria					
mechanism					
fall > 3 m	5	20.8	0	0.0	0.001
ejection out of a vehicle	0	0.0	0	0.0	1.0
geriatrics: fracture of long bone	1	4.2	13	13.0	0.3
geriatrics: ≥2 injured regions	4	16.7	11	11.0	0.49
injury pattern					
instable chest	2	8.3	0	0.0	0.04
amputation (excl. finger/toe)	0	0.0	0	0.0	1.0
instable pelvis	1	4.2	0	0.0	0.18
≥2 fracture proximal long bone	2	8.3	0	0.0	0.04
≥20% burning IIb°/III°	0	0.0	0	0.0	1.0
bad feeling	5	20.8	0	0.0	0.001
Stab and gunshot wounds to the head/neck/torso	2	8.3	0	0.0	0.04

Key: NIV = non-invasive ventilation, GCS = Glasgow Coma Scale, NIBD = non-invasive blood pressure, STEMI = ST segment elevation myocardial infarction; categorical variables were analyzed using Fisher’s test.

## Data Availability

The datasets used and/or analyzed during the current study are available from the corresponding author on reasonable request.

## Abbreviations

The following abbreviations are used in this manuscript:

ABCD	Airway, Breathing, Circulation, Disability (primary assessment scheme)
CI	confidence interval
CPU	chest pain unit
CVC	central venous catheter
CT	computed tomography
ED	emergency department
eCV	electrical cardioversion
ETI	endotracheal intubation
GCS	Glasgow Coma Scale
ICU	intensive care unit
LR+/− LR	Positive/Negative Likelihood Ratio
M^2^-GRETA	Modified Moenchengladbach Resuscitation Team Activation
MRI	magnetic resonance imaging
NIBD	non-invasive blood pressure
NIV	non-invasive ventilation
PPV/NPV	positive/negative predictive value
SD	standard deviation
STEMI	ST segment elevation myocardial infarction
V_2_iSiOn	Vital Sign–Based Alert Criteria (non-trauma)
